# Comparative study between Fenton and intergrowth 21 charts in a sample of Lebanese premature babies

**DOI:** 10.1186/s12887-020-1968-7

**Published:** 2020-02-17

**Authors:** Marie Samarani, Gianna Restom, Joelle Mardini, Georges Abi Fares, Souheil Hallit, Marie-Claude Fadous Khalife

**Affiliations:** 1 Faculty of Medicine and Medical Sciences, Holy Spirit University of Kaslik (USEK), Jounieh, Lebanon; 2 Pediatrics Department, Notre Dame Des Secours University Hospital, Byblos, Lebanon; 3 INSPECT-LB: Institut National de Sante Publique, Epidemiologie Clinique et Toxicologie, Beirut, Lebanon

**Keywords:** Growth charts, Percentiles, Premature, Fenton, Intergrowth-21

## Abstract

**Background:**

Different charts are used to assess premature growth. The Fenton chart, based on prenatal growth, has been used in the neonates’ intensive care unit (NICU) of the Notre Dame des Secours University Hospital to assess premature newborns’ development. Intergrowth21 is a new multidisciplinary, multiethnic growth chart better adapted to premature growth. Our objective was to compare both charts Fenton and Intergrowth21 in order to implement Intergrowth in our unit.

**Methods:**

We analyzed 318 files of premature babies born who were admitted to the NICU from 2010 till 2017. Anthropometric data (weight, height and head circumference) converted to percentiles was filled on both charts from birth till 1 month of age.

**Results:**

The results of the linear regression, taking the weight at birth as the dependent variable, showed that the Fenton scale (R2 = 0.391) would predict the weight at birth better than the Intergrowth 21 scale (R2 = 0.257). The same applies for height and cranial perimeter at birth when taken as dependent variables. When considering the weight and height at 2 weeks, the results showed that the Intergrowth 21 scale would predict those variables better than the Fenton scale, with higher R2 values higher in favor of the Intergrowth 21 scale for both weight (0.384 vs 0.311) and height (0.650 vs 0.585). At 4 weeks, the results showed that the Fenton scale would predict weight (R2 = 0.655 vs 0.631) and height (R2 = 0.710 vs 0.643) better than the Intergrowth 21 scale. The results obtained were adjusted over the newborns’ sociodemographic and clinical factors.

**Conclusion:**

The results of our study are controversial where the Fenton growth charts are superior to Intergrowth 21 before 2 weeks of age and at 4 weeks, whereas Intergrowth 21 charts showed higher percentiles for weight and height than Fenton charts at 2 two weeks of age. Further studies following a different design, such as a clinical trial or a prospective study, taking multiple ethnicities into account and conducted in multiple centers should be considered to enroll a more representative sample of Lebanese children and be able to extrapolate our results to the national level.

## Introduction

Prematurity is becoming more frequent nowadays especially with the development of artificial fertilization methods [1]. In 2016, the Center for Disease Control and Prevention (CDC) declared that around one baby out of 10 is born premature [2, 3]. Newborn’s growth is an important marker and a screening method for a number of pathologies or deficiencies [4], which needs to be tracked through growth charts. The latter would lead to a better monitoring of the nutritional status, thus, may limit the depth and duration of diet-related growth restriction and its short- and long-term damages thereafter [3].

In fact, many charts have been developed, mostly based on intrauterine growth and rarely adapted to preterm newborns. Indeed, preterm babies are not fetuses as they no longer live in-utero [ 5]. Regardless of their apparent independence, they have not acquired the growth and survival skills of full-term babies yet and present a physiological immaturity. Consequently, when assessed via common growth charts, these newborns remain under the 10th percentile for a long time and do not catch up with normal growth until the age of two to three years. For this motive, the actual trend is to supplement this population with a hypercaloric nutrition to compensate for this extra-uterine growth restriction. Despite this supplementation, most babies fail to reach their set growth goals still.

Within that scope, alarming studies have shown an association between prematurity and obesity in adulthood, with question marks raised about the link between “overfeeding” the preterm newborns, obesity and cardiovascular complications later in life [6]. In the neonatal population aged between 36 and 50 weeks of unadjusted age, the Fenton chart is considered one of the best charts for assessing longitudinal growth [7]. Nevertheless, it showed two weaknesses: it does not reflect the adaptation of the premature newborn to extra-uterine life and it under- or overestimates newborn’s growth.

The most commonly used chart at the Notre Dame des Secours University Hospital Center-Byblos (CHU-NDS), is the Fenton chart 2003, which has not been updated till now. Between 2009 and 2014, the Intergrowth21 project has emerged as a successful growth chart and underwent rigorous processes that ensured that the data collected in the INTERGROWTH-21st project is of exceptionally high quality [8]. Intergrowth-21 charts are used to create standards for postnatal growth of premature infants especially those born before 32 gestational weeks [9]. While disagreements on the Fenton charts continue, the results of the Intergrowth 21st project were awaited with great interest. The “Intergrowth 21^st^ Project” was a prospective multicenter, multi-ethnic study, which included low-risk women, non-smokers, with a normal pregnancy history, and no health problems that could affect fetal growth [10]. All maternal health care and nutritional needs were met. Birth and postnatal growth standards were developed from data collected from a cohort of uncomplicated pregnancies with normal growing fetuses [11]. These very strict selection criteria were mandatory, in order to create standards on how the normal growth of healthy premature babies should be.

In a recent systematic review, 61 longitudinal reference charts were identified and compared to the Intergrowth-21 chart [9]; assessments made using the Intergrowth-21 charts demonstrated a reduction in the diagnosis of extrauterine growth retardation [9, 12]. Many infants who were classified as having restricted growth according to the Fenton charts, turned out to have normal postnatal growth according to the Intergrowth-21 charts [12]. Another important point is that, like the World Health Organization (WHO) growth standards, the Intergrowth-21 growth standards aim at producing graphs that describe optimal rather than average growth, which could be used worldwide.

Being in a developing country, a local validation before adapting Intergrowth-21 charts to our new born infants is necessary, especially to avoid the misclassification of their size, which may have an impact on their nutritional support. For these reasons, the objective of this study was to check which method (the universal Fenton 2003 curves or the Intergrowth-21 curves) used in the neonatology department at CHU-NDS would predict height, weight and cranial perimeter of premature Lebanese babies better. This study would help us evaluate the difference between both curves in terms of extra- and intra-uterine growth restriction, reflected by weight, height and head circumference at birth and verify later the convergence between the intergrowth-21 and the WHO curves of the child health record book around the sixth month of life.

## Methods

### Study design

This was a retrospective study, conducted at CHU-NDS. Medical records of premature newborns admitted to the neonatal unit over a seven-year period (2010 to 2017), were reviewed. The discretion of names and personal information have been respected. All preterm infants born alive before 37 weeks of gestation and admitted to the neonatology department within 24 h of birth, were included in the study. Term infants (born at 37 weeks of gestation or more) were excluded since Intergrowth-21 is a growth chart adapted only to preterm babies. Furthermore, excluded were [1] newborns admitted after 24 h of birth to the neonatal intensive care unit (NICU) [2], who died during hospitalization [3], who were transferred to another hospital and [4] who were suffering from a comorbidity that can affect normal growth, such as bronchodysplasia, cardiovascular pathologies and placental insufficiency or any other prenatal diseases known to alter the normal pattern of growth.

### Data collection

Data was collected from files in the medical archive. Weight, height and head circumference of each child at birth, at 37 weeks of gestation, 2 weeks and 4 weeks of life were noted, and then marked on the percentile curves of the Fenton 2003 and Intergrowth 21 charts. Weight and height were measured using a digital baby scale with a rod, whereas head circumference was obtained via a measuring tape; the same measurement method was followed for all children. The follow-up data of each child after discharge were also collected from medical records of each child’s pediatrician.

When the measurements fell on the curves between 2 standard lines of percentiles, the value was then approximated to an intermediate value between the two percentiles. Thus, the 5th, 30th, 70th and 95th percentile were considered if the measurements fell between the following brackets 3rd-10th, 10th–50th, 50th–90th and 90th–97th percentile respectively. Values ​​below the 3rd percentile or above the 97th percentile were reported as 2nd and 98th percentile respectively. This approximation was made for both charts in order to avoid any bias.

The data collection took into account other variables such as the date of birth of the new born, the length of stay at the hospital, the need for intubation, transfusion, iron supplementation, the cause of admission to the NICU, consanguinity, medically assisted procreation (In-vitro fertilization-IVF) and the delivery method.

### Statistical analysis

Statistical analysis of data was performed using SPSS version 22 (SPSS Inc., Chicago, IL, USA). Comparisons of the same baby’s measures according to both charts were assessed through linear regressions. Multiple linear regressions were conducted taking weight, height and cranial perimeter as dependent variables and taking in each model one of the charts as an independent variable. The model that had a higher Nagelkerke R^2^ value would predict the dependent variable more.

## Results

**Fig. 1:**
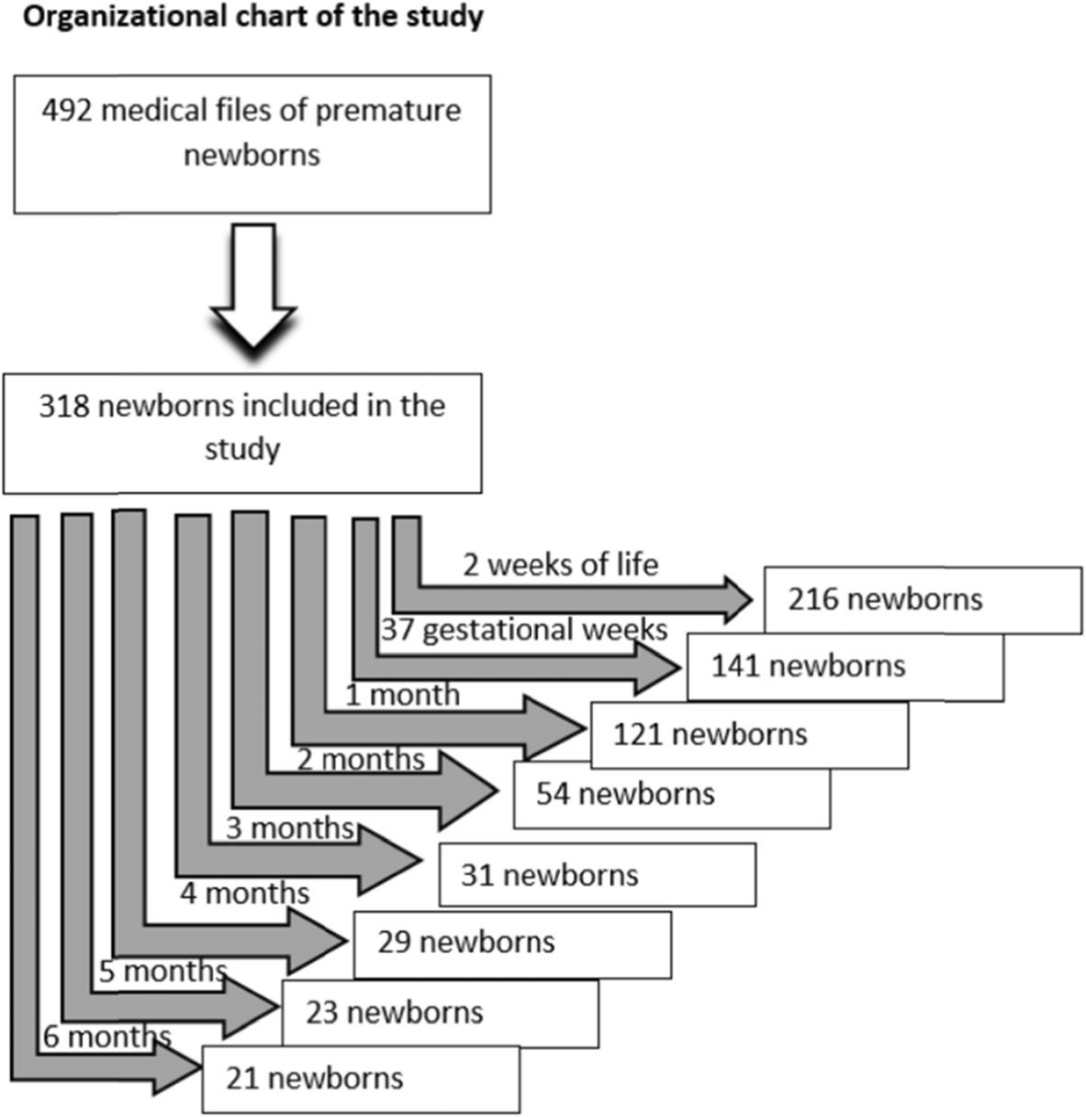
Organizational chart of the study

Out of a total of 492 medical record extracted, 318 (64.63%) newborns aged between 27 and 36 weeks of gestation met the inclusion criteria. The distribution of gestational ages showed that 52.8% of the babies were born between 34 and 36 gestational weeks, whereas the remaining newborns were under 33 gestational weeks (Fig. 1). The most frequent cause of admission to the NICU was multiple pregnancies (32.4%), followed by placental insufficiency (22%), respiratory distress of different etiologies (22%) and infections (20.1%).

The majority of the newborns (98.4%) were admitted to the NICU of the CHU-NDS from maternity ward and 1.6% were transferred before birth from another hospital. The mean age of birth was 33.26 ± 2.10 weeks of gestation. Consanguinity was present in 11.6% of the cases and caesarean section accounted for 85.8% of deliveries. Moreover, 29.9% of the babies were intubated and 78.8% received more than 2 blood transfusions during their stay; 49.7% of infants were fed by breast milk and formula milk, 48.1% by formula milk alone and 1.3% were exclusively breastfed. We note that in-vitro fertilization methods accounted for 24.8% of pregnancies.

### Difference between the two charts

The results of the linear regression taking weight at birth as the dependent variable, showed that the Fenton scale (R^2^ = 0.391) would predict weight at birth better than the intergrowth 21 scale (R^2^ = 0.257) (Table 1, Model 1). The same applies for height (Table 1, Model 2) and cranial perimeter (Table 1, Model 3) at birth when taken as dependent variables. In contrast, when considering weight and height at 2 weeks, the results showed that the Intergrowth-21 chart would predict weight (0.384 vs 0.311) (Table 1, Model 4) and height (0.650 vs 0.585) (Table 1, Model 5) more than the Fenton chart. When considering weight and height at 4 weeks, the results showed that the Fenton chart would predict weight (R2 = 0.655 vs 0.631) and height (R2 = 0.710 vs 0.643) better than the Intergrowth-21 chart (Table 1, Models 6 and 7 respectively).

**Table 1:** Linear regressions of factors associated with the baby’s parameters at birth according to the Fenton and Intergrowth 21 charts

**Model 1: Dependent variable: Weight at birth.**								
		**Fenton scale**			**Intergrowth 21 scale**			
**Variable**	**Unstandardized Beta**	***p*-value**	**Confidence Interval**		**Unstandardized Beta**	**p-value**	**Confidence Interval**	
Intubation	−196.278	.001	− 313.717	−78.839	− 145.980	.027	− 275.295	− 16.666
Gender (females vs males*)	− 157.258	.004	−263.479	−51.038	− 140.141	.020	− 258.511	− 21.770
In-vitro fertilization (yes vs no*)	− 398.452	< 0.001	− 521.357	− 275.548	− 437.977	< 0.001	− 573.322	−302.631
Delivery method (C-section vs normal*)	− 19.570	.793	− 166.432	127.291	17.286	.836	− 147.337	181.910
Any cause of prematurity (yes vs no*)	20.384	.378	−25.087	65.855	−8.373	.740	−58.030	41.283
Consanguinity (yes vs no*)	37.530	.642	− 121.321	196.381	85.564	.337	−89.577	260.705
Breastfeeding (yes vs no*)	33.230	.068	−2.498	68.958	42.203	.036	2.695	81.712
	R2 = 0.391				R2 = 0.257			
**Model 2: Dependent variable: Height at birth.**								
		**Fenton scale**			**Intergrowth 21 scale**			
**Variable**	**Unstandardized Beta**	**p-value**	**Confidence Interval**		**Unstandardized Beta**	**p-value**	**Confidence Interval**	
Intubation (yes vs no*)	−1.053	.010	−1.855	−.251	−1.188	.006	−2.036	−.341
Gender (females vs males*)	−.993	.008	−1.730	−.256	−.575	.147	−1.353	.203
In-vitro fertilization (yes vs no*)	−1.604	< 0.001	−2.494	−.715	−1.573	.001	−2.506	−.641
Delivery method (C-section vs normal*)	−.301	.551	−1.295	.694	−.388	.463	−1.431	.654
Breastfeeding (yes vs no*)	.144	.249	−.101	.389	.143	.273	−.114	.401
Any cause of prematurity (yes vs no*)	.080	.601	−.223	.383	.081	.616	−.238	.400
Consanguinity (yes vs no*)	−.075	.895	−1.201	1.050	.332	.578	−.842	1.505
Length percentile at birth	.065	< 0.001	.052	.078	.053	< 0.001	.040	.065
	R2 = 0.368				R2 = 0.305			
**Model 3: Dependent variable: Cranial perimeter at birth.**								
		**Fenton scale**			**Intergrowth 21 scale**			
**Variable**	**Unstandardized Beta**	**p-value**	**Confidence Interval**		**Unstandardized Beta**	**p-value**	**Confidence Interval**	
Intubation (yes vs no*)	.391	.021	.060	.723	.194	.287	−.165	.554
Gender (females vs males*)	−.237	.127	−.542	.068	.474	.010	.113	.836
In-vitro fertilization (yes vs no*)	−.183	.311	−.537	.172	−.071	.720	−.461	.319
Delivery method (C-section vs normal)	.012	.955	−.409	.433	.031	.896	−.428	.489
Any cause of prematurity (yes vs no*)	.076	.247	−.053	.205	.032	.656	−.109	.172
Consanguinity (yes vs no*)	−.008	.972	−.478	.462	.018	.943	−.493	.530
Breastfeeding (yes vs no*)	.009	.865	−.094	.112	.069	.226	−.043	.180
Head circumference at birth	.042	.000	.037	.047	.035	.000	.030	.040
	R2 = 0.498				R2 = 0.405			
**Model 4: Dependent variable: Weight at 2 weeks.**								
		**Fenton scale**			** Intergrowth 21 scale**			
**Variable**	**Unstandardized Beta**	**p-value**	**Confidence Interval**		**Unstandardized Beta**	**p-value**	**Confidence Interval**	
Intubation (yes vs no*)	− 164.040	.010	−287.562	−40.517	− 178.365	.003	−295.261	−61.469
Gender (females vs males*)	− 111.614	.063	− 229.505	6.276	−35.214	.546	− 149.985	79.556
In-vitro fertilization (yes vs no*)	−353.688	.000	− 487.945	− 219.430	− 363.595	.000	− 490.281	− 236.909
Delivery method (C-section vs normal*)	−9.015	.917	− 179.313	161.283	9.309	.909	−151.824	170.442
Any cause of prematurity (yes vs no*)	36.339	.171	−15.846	88.525	47.720	.059	−1.849	97.288
Consanguinity (yes vs no*)	22.674	.801	− 154.905	200.254	44.992	.597	− 122.662	212.646
Breastfeeding (yes vs no*)	−.757	.970	−40.738	39.223	−1.103	.954	−38.889	36.683
Weight percentile at 2 weeks of age	11.378	.000	7.927	14.830	11.141	.000	8.496	13.785
	R2 = 0.311				R2 = 0.384			
**Model 5: Dependent variable: Height at 2 weeks.**								
		**Fenton scale**			**Intergrowth 21 scale**			
**Variable**	**Unstandardized Beta**	**p-value**	**Confidence Interval**		**Unstandardized Beta**	**p-value**	**Confidence Interval**	
Intubation (yes vs no*)	.673	.230	−.448	1.794	.771	.128	−.235	1.777
Gender (females vs males*)	−.579	.239	−1.562	.403	−.136	.769	−1.066	.795
In-vitro fertilization (yes vs no*)	−1.872	.028	−3.528	−.217	−1.697	.029	−3.209	−.185
Delivery method (C-section vs normal*)	−.984	.149	−2.339	.370	−.704	.261	−1.955	.547
Any cause of prematurity (yes vs no*)	−.129	.570	−.588	.329	−.060	.770	−.474	.354
Consanguinity (yes vs no*)	1.678	.059	−.070	3.426	1.177	.143	−.418	2.772
Breastfeeding (yes vs no*)	−.161	.335	−.495	.173	−.149	.331	−.455	.158
Length percentile at 2 weeks	.058	.000	.037	.079	.054	.000	.037	.070
	R2 = 0.585				R2 = 0.650			
**Model 6: Dependent variable: Weight at 4 weeks.**								
		**Fenton scale**			**Intergrowth 21 scale**			
**Variable**	**Unstandardized Beta**	**p-value**	**Confidence Interval**		**Unstandardized Beta**	**p-value**	**Confidence Interval**	
Intubation (yes vs no*)	− 349.864	.000	−511.389	−188.338	−287.552	.001	− 456.257	− 118.846
Gender (females vs males*)	−214.487	.006	− 366.769	−62.205	− 104.602	.204	− 266.918	57.714
In-vitro fertilization (yes vs no*)	−263.235	.003	−433.325	−93.146	− 316.056	.000	−490.141	−141.971
Delivery method (C-section vs normal*)	−49.404	.659	− 270.890	172.083	−101.449	.380	−329.711	126.814
Any cause of prematurity (yes vs no*)	60.329	.079	−7.030	127.689	96.435	.008	25.542	167.329
Consanguinity (yes vs no*)	72.829	.508	−144.660	290.318	78.345	.491	−146.491	303.181
Breastfeeding (yes vs no*)	−10.813	.692	−64.785	43.160	−13.781	.625	−69.600	42.038
Weight percentile at 4 weeks	21.310	.000	17.484	25.136	18.974	.000	15.344	22.604
	R2 = 0.655				R2 = 0.631			
**Model 7: Dependent variable: Height at 4 weeks.**								
		**Fenton scale**			**Intergrowth 21 scale**			
**Variable**	**Unstandardized Beta**	**p-value**	**Confidence Interval**		**Unstandardized Beta**	***p*-value**	**Confidence Interval**	
Intubation (yes vs no*)	−1.278	.071	−2.668	.113	−.553	.465	−2.065	.959
Gender (females vs males*)	−1.068	.055	−2.161	.024	−.559	.363	−1.784	.665
In-vitro fertilization (yes vs no*)	−1.518	.016	−2.734	−.302	−1.096	.121	−2.493	.300
Delivery method (C-section vs normal*)	−.298	.703	−1.859	1.264	−1.735	.053	−3.494	.025
Any cause of prematurity (yes vs no*)	−.215	.364	−.689	.258	−.259	.325	−.783	.265
Consanguinity (yes vs no*)	.317	.748	−1.660	2.294	1.860	.086	−.277	3.996
Breastfeeding (yes vs no*)	−.127	.513	−.516	.262	−.149	.490	−.580	.282
Length percentile at 4 weeks	.100	.000	.077	.124	.078	.000	.056	.100
	R2 = 0.710				R2 = 0.643			

## Discussion

Growth monitoring is an essential tool that reflects the overall health of neonates, especially preterm infants. It helps assess the nutritional status and detect pathological deviations. A meta-analysis, published in 2015, of 16 prospective cohorts of premature newborn comparing the 1991 US birthweight reference, the 1999–2000 US birthweight reference and the Intergrowth-21st standards, revealed a prevalent reduction of small for gestational age preterm newborn by more than a quarter, with no significant change in the risk of associated neonatal mortality [13]. Conversely, newer results from a retrospective study showed that the incidence of small for gestational age preterm newborns was higher with the Intergrowth 21st standards compared to the Fenton ones. The difference between the results of those research [12] prompted us to conduct our study. Growth curves monitor height, weight, and head circumference progression, therefore a reference chart adopting growth curves that are applicable for all ethnicities and races using anthropometric measures should be used in order to provide adequate assessment [14]. In our study, a comparison of the weight and height percentiles of the whole sample showed that before two weeks of age, Fenton growth charts showed better results compared to the Intergrowth 21; after two weeks of age, Intergrowth 21 charts showed higher R2 values for weight and height than Fenton charts.

The Fenton 2003 growth charts have been adopted in the NICU of the CHU-NDS so far in order to follow the improvement of growth in preterm neonates, especially those receiving parenteral nutrition according to the international nutritional guidelines. In most cases, these curves have shown these infants to have growth retardation despite adequate nutrition and introduction of amino acids, electrolytes and multivitamin complexes very early; consequently, those babies are exposed to intensive parenteral nutrition for a long period of time, which further delays their discharge from NICU. The main reason behind this is that Fenton growth charts assessment is based on intrauterine growth standards [15], causing the overfeeding of these newborns to lead to obesity and metabolic syndrome later in life. On another hand, the Intergrowth-21 standards aimed at producing charts that set breastfeeding as the norm to follow and described optimal rather than average growth, which could be used worldwide [ 16].

### Study limitations

Our sample data was difficult to collect after hospital discharge since pediatricians do not keep records of their patients’ growth in their offices and rely on medical files kept by the parents. Our study is retrospective that predisposes us to an information bias since we didn’t get the chance of collecting all the data we need from some files. Plus, the effect of the maternal height and weight on the results was not studied and should have been investigated since increased maternal height and weight are correlated with increased infant’s birth weight. Future studies that follow a different design (clinical trial or prospective) should be considered to avoid the bias in anthropometric measurements. A more representative sample of Lebanese children recruited from multiple centers is warranted to extrapolate the results to the whole population. Finally, prenatal diseases that could alter the pattern of growth should be taken into consideration.

## Conclusion

The results of our study are controversial since the Fenton growth charts showed superiority predicting newborn’s growth in terms of weight, height and cranial perimeter at birth and at 4 weeks compared to the Intergrowth-21 ones, whereas Intergrowth 21 charts showed higher percentiles for weight and height at 2 two weeks of age compared to the Fenton charts. The results obtained could have been affected by many factors, including ethnicity that could not be investigated in this study due to its retrospective aspect. Therefore, further studies that take this study’s limitations into account, are needed.

## Data Availability

There is no public access to all data generated or analyzed during this study to preserve the privacy of the identities of the individuals. The dataset that supports the conclusions is available to the corresponding author upon request.

## Abbreviations

CDC: Center for Disease Control and Prevention
CHU-NDS: Notre Dame des Secours University Hospital Center-Byblos
IVF: In-vitro fertilization
NICU: neonatal intensive care unit
WHO: World Health Organization
